# A Digital Tool for Assessing Well-Being at the Workplace and in Personal Life: Development and Validation of the Quan Well-Being Index

**DOI:** 10.2196/73713

**Published:** 2025-08-08

**Authors:** Georgia A Floridou, Freya Katre, Emile Jeuken

**Affiliations:** 1 Quan The Hague The Netherlands; 2 King’s Business School King’s College London London United Kingdom

**Keywords:** workplace well-being, personal life well-being, index development, digital health

## Abstract

**Background:**

Quan is a workplace well-being digital platform that supports employees, teams, and organizations in measuring, understanding, and improving their well-being. It is important to develop a validated measurement instrument that enables users to assess and track their well-being over time. Currently, no digital measurement instrument comprehensively evaluates well-being across both personal and professional domains.

**Objective:**

We detail the development and validation of the Quan Well-being Index, a new digital self-report measure for assessing well-being in personal life and at the workplace.

**Methods:**

We performed 3 studies. The first study involved the conceptualization of 6 initial factors, the generation of 51 items, and the steps of face and content validity. In the second study, revised items were presented to a UK sample. In the third study, an independent UK sample completed the final assessment along with a battery of well-being and personality questionnaires. A subsample of participants from the third study retook the assessment approximately 2 weeks after initial completion.

**Results:**

In the first study, after face and content validity processes, the number of items was reduced to 45. In the second study, exploratory factor analysis on data from 1020 participants (age: mean 43.06, SD 12.98 years; 525 female participants) identified a 4-factor solution with 35 items (Kaiser-Meyer-Olkin value=0.98; Bartlett test: *χ*^2^_990_=37063.54; *P*<.001), accounting for 64% of variance. The 4 factors were *thrive and connect in personal life*, *thrive and connect at work*, *mental health*, and *physical health*. In the third study, confirmatory factor analysis on data from 966 participants (age: mean 44.4, SD 12.52 years; 480 female participants) tested 4 structural models. A hierarchical model (model 1) where the general factor influenced the 4 group factors demonstrated the best fit (*χ*^2^_521_=3467.00; Bentler comparative fit index=0.906; Tucker-Lewis index=0.892; root mean square error of approximation=0.077; standardized root mean square residual=0.048; ΔAkaike information criterion=0.0; ΔBayesian information criterion=0.0). Internal reliability was high across subscales (Cronbach α=.88-.93; McDonald ω total=0.89-0.94; Guttman λ6=0.86-0.92). Convergent validity was demonstrated by strong correlations with the Warwick-Edinburgh Mental Well-being Scale (*r*=0.45-0.85; *P*<.001) and Flourishing-at-Work Scale (*r*=0.80-0.84; *P*<.001). Divergent validity was supported through weak or negative correlations with Big Five Personality Inventory traits (eg, neuroticism: *r*=–0.29; *P*<.001). Test-retest reliability assessed in a subset of 275 participants (age: mean 52.12, SD 9.56 years; 170 female participants) over a 2-week interval was strong to very strong across factors (*r*=0.74-0.81; *P*<.001).

**Conclusions:**

The Quan Well-being Index provides a comprehensive assessment of well-being at the workplace and in personal life, and is anticipated to be a valuable digital tool, enabling individuals, teams, and organizations to gain insights, monitor progress, and implement appropriate interventions for a healthier workforce.

## Introduction

### Background

Well-being has become a pressing concern in the modern workplace, with stress, burnout, and other issues on the rise. Evidence suggests that well-being issues can result in reduced productivity, increased absenteeism, and medical expenses for organizations [1,2], as well as impact teamwork, decision-making, and innovation [3]. By addressing well-being in the workplace, organizations can create a healthier culture and work environment, which can result in increased employee engagement, performance, and productivity, as well as better financial outcomes and retention [4]. Moreover, fostering workplace well-being can have broader societal benefits, such as reducing health care burdens, promoting mental health awareness, and contributing to overall community well-being [5-7]. Therefore, it is crucial to prioritize well-being in the workplace, and digital health tools offer promising solutions for achieving this goal.

In recent years, there have been calls for digital health implementation in workplace settings [8]. Although the digital health market is thriving, with over 350,000 products available and a growth rate of 25% per year [9], digital health technologies for the workplace are minimal, and existing ones focus on employees at the individual level and do not address team and organizational workplace issues [10]. Recognizing the need for digital health interventions, the World Health Organization [11] and the European Commission [12] have published guidelines for digital health tools, with the main objective of evidence-based practice. A key problem with existing digital health products is that the regulation around them is minimal [13]. The majority of these products do not always reach the basic standards of scientific rigor, such as peer-reviewed publications, with only 2.08% being evidence-based [14]. Since traditional approaches, such as employee assistance programs, are challenging to reach and have low uptake [15] and remote and hybrid work is rising, there is a need for evidence-based digital health solutions in the workplace.

According to Stevenson and Farmer [8], well-being measurement is a crucial workplace core standard for employees and teams to monitor well-being and take positive action to improve it. It also enables team leads and organizations to use the results to establish appropriate well-being strategies and enhance organizational well-being in general. Therefore, there is a crucial need for a robust well-being measurement instrument in the workplace for employees, teams, and organizations. One platform that aims to fulfill the need for such solutions is Quan, a digital well-being platform for employees, teams, and organizations, which has a 3-fold process: (1) quantify well-being at employee, team, and organizational levels; (2) provide comprehensive and personalized well-being insights as well as information on the underlying reasons; and (3) deliver tailored interventions for individual employees and teams. Our research is the first step toward this approach and concerns the development of a novel, valid, and reliable measurement instrument that assesses well-being at the workplace and in personal life. This instrument is called the Quan Well-being Index (QWI).

### Assessment of Well-Being at the Workplace and in Personal Life

The development of a novel, valid, and reliable measurement instrument for the well-being of employees, teams, and organizations, which will be used in a digital setting, is of pivotal importance. Currently, no measurement instrument assesses well-being in both personal and work life. A plethora of measures exist for assessing various well-being aspects of personal life. Despite the growth of self-report well-being questionnaires, there is significant variation in their conceptualization of well-being (eg, eudemonic and hedonic well-being, and quality of life) and their methodological aspects such as length and psychometric properties [16]. Furthermore, the majority of available measures address well-being in general or in personal life (eg, the Warwick-Edinburgh Mental Well-being Scale [17]) or focus on only 1 aspect, such as quality of life (eg, Quality of Life Inventory [18]). Moreover, the majority of well-being measures prioritize mental well-being over aspects related to physical health, such as sleep, nutrition, and exercise. On the other hand, the assessment of well-being in domain-specific settings, such as work, is still in its infancy, with only a few existing validated measures [19-21] that capture only workplace-related well-being such as job satisfaction, burnout, and stress [22-24]. To establish a comprehensive model of occupational well-being, work-related and personal facets need to be considered [25].

### Frameworks for Well-Being in the Workplace

Various theoretical models have been developed to explain the factors that contribute to well-being in the workplace, with particular emphasis on the interaction between job characteristics and psychological needs. Among these, the Job Demands-Resources (JD-R) model and Self-Determination Theory (SDT) have emerged as 2 of the most influential frameworks, offering complementary perspectives on how workplace conditions and intrinsic motivation shape well-being. The JD-R model [26] stands out as the primary framework for conceptualizing the drivers of employee well-being, and its effectiveness is, in part, attributed to its suitability in interpreting interventions within the workplace [27-29]. According to the JD-R model, the mental well-being of workers results from the interplay of 2 distinct categories: job demands and job resources. Job demands encompass the various facets of work, such as physical, psychological, social, or organizational, that necessitate sustained effort, whether it be physical, cognitive, or emotional [27]. Job resources refer to the physical, psychological, social, or organizational elements of work that have the potential to motivate, assist in achieving work-related goals, mitigate the effects of job demands, and foster personal development and learning [27]. Beyond workplace factors, the JD-R model also acknowledges the role of personal demands and resources in shaping well-being. Personal demands refer to individual characteristics or life circumstances, such as caregiving responsibilities, financial stress, and chronic health conditions, that require effort and may contribute to strain [30]. Conversely, personal resources, including resilience, self-efficacy, optimism, and emotional intelligence, can help employees cope with challenges, enhance motivation, and buffer the negative effects of high job demands [30]. The interaction between job-related factors and personal factors is crucial, as personal resources can amplify the benefits of job resources, while personal demands may exacerbate workplace stressors, further influencing overall well-being.

The SDT has emerged as a crucial framework for understanding and promoting well-being in the workplace. Grounded in the work of Ryan and Deci [31], the SDT highlights the fundamental psychological needs of autonomy, competence, and relatedness, asserting that meeting these needs is essential for ideal human functioning and well-being [31]. In the workplace, the SDT has been instrumental in explaining the impact of autonomy on employee motivation and satisfaction [32]. When individuals perceive a high degree of autonomy in their work, they could have increased job satisfaction and engagement [33]. Furthermore, the fulfillment of competence needs, achieved through opportunities for skill development and feedback, is linked to enhanced job performance and personal well-being [34]. Finally, creating a workplace environment that supports relatedness and fosters positive social connections and interpersonal relationships has been linked with increased levels of well-being and job satisfaction [35]. Thus, the application of the SDT in the workplace not only contributes to individual well-being but also holds promise for enhancing organizational outcomes. However, so far, no measurement instrument of workplace and personal life well-being has considered the JD-R model and the SDT. Therefore, there is a need for a novel, holistic, and robust measurement instrument that takes into consideration these frameworks and simultaneously assesses well-being at the workplace and in personal life, as these settings are intricately associated.

### Objectives of the QWI

The development of a digital workplace and personal life well-being index will support employees at the individual level to track, reflect, act on, and ultimately improve their well-being. Existing evidence suggests that digital mental health platforms that provide measurement scores to their users may improve their mental health by supporting them to be aware of and reflect on it [36,37]. At the team level, the index will act as a catalyst for establishing bidirectional well-being communication between employees and their team leads, which has been shown to contribute to improved well-being and increased trust between them [38]. Furthermore, such a measurement instrument will support users in assessing and monitoring their well-being across time by identifying areas of strength and risk and subsequently the impact of tailored interventions. Finally, at the organizational level, the index will provide organizations with metrics that will help them identify which well-being initiatives they should invest in and offer to their employees. This, in turn, will lead to improved employee and team well-being and better functioning organizations.

### Overview of Studies

The primary objective of this project is to create and validate a comprehensive self-report tool called the QWI. This instrument aims to evaluate the complete range of well-being aspects in both professional and personal domains for individuals, teams, and organizations. Building upon existing well-being theories and models, this research encompasses 3 studies focused on developing and refining the QWI using a British sample. Study 1 details the creation of the initial item pool and the process to ensure face and content validity. Study 2 describes the development of the QWI, using data collected from online panels to explore the factor structure. Study 3 employs a separate sample to verify the structure of the QWI and examines various models using a confirmatory approach. Additionally, study 3 reports on the internal reliability, convergent and divergent validity, and test-retest reliability of the QWI through psychometric indicators.

### Aim

The aim is to develop and validate a novel, psychometrically robust self-report instrument called the QWI, which is designed to assess well-being across both the workplace and personal life domains. The QWI seeks to capture individual, team, and organizational well-being to support digital health applications in workplace settings.

## Study 1

### Methods

#### Item Generation

The initial item pool was generated by an experienced organizational psychologist (EJ), who has over 15 years of practice in academia and occupational health psychology, leadership development, and employee well-being consultation across diverse industries. The item development was informed by a combination of applied practice and an integrative review of the literature on personal life and workplace well-being, including key constructs from the JD-R model [26] and SDT [31]. The JD-R model posits that well-being at work is shaped by a balance between job demands (eg, workload and emotional strain) and job resources (eg, autonomy and social support). This model provided the foundation for generating items throughout the initial model, ensuring that both protective and risk-related aspects of the work environment were represented. The SDT highlights the importance of fulfilling basic psychological needs, such as autonomy, competence, and relatedness, that are essential for well-being. Additional well-being models, such as PERMA (positive emotion, engagement, relationships, meaning, and accomplishment) [39] and Keyes’ model of flourishing [40], also informed item generation, particularly for capturing positive functioning beyond symptom reduction. These frameworks collectively informed the conceptual structure of the measure.

Based on this theoretical foundation, a preliminary set of 51 items was generated, each intended to capture an aspect of individual well-being relevant in the personal life and workplace contexts. The items were grouped into emergent and coherent themes based on conceptual similarity, resulting in 6 factors, which formed the foundation of our well-being definition (ie, the experience of mental and physical health and the sense of meaning, self-fulfillment, and social connectedness). These were labeled as: *mind* (11 items), *body* (8 items), *social connectedness* (8 items), *meaning* (10 items), *self-fulfillment* (11 items), and *general well-being* (3 items). These factors collectively formed the initial conceptual framework for the QWI. This process ensured that the QWI is grounded in theory, responsive to practical workplace realities, and reflective of multidimensional well-being that spans both personal and professional settings.

We acknowledge that relying on a single expert for the item generation and thematic grouping is a limitation, particularly regarding potential bias and limited perspective. However, subsequent phases of the project, including expert review and empirical validation (described in the face and content validity subsections and in studies 2 and 3), were used to mitigate this limitation and refine the structure and content of the QWI.

#### Face Validity

We presented the 51 items to 4 English-speaking nontechnical staff members at Quan (2 males, 1 female, and 1 nonbinary) with a mean work experience of 16 (range 1-25) years and individually asked them to assess the items by rating them on how clear and understandable they are (1=not clear and understandable, 2=somewhat clear and understandable, 3=clear and understandable, and 4=very clear and understandable) and to provide qualitative feedback on what is unclear and not easily understandable for items rated as 1 or 2 and provide suggestions on rewording.

#### Content Validity

We asked 4 experts in the field of well-being (2 academic psychologists and 2 practitioners; 2 females and 2 males) with a mean professional experience of 15.6 (range 10-20) years to rate the 51 items on how well they assessed each construct (1=not relevant, 2=somewhat relevant, 3=quite relevant, and 4=highly relevant) and to provide qualitative feedback and suggestions for rewording or removing items.

#### Ethical Considerations

##### Human Subject Ethics Review Approvals or Exemptions

All studies were reviewed and approved by the Ethics Board of King’s Business School, King’s College London, under reference number HR/DP-22/23-34006. The research involved human participants, and all procedures were conducted in accordance with the ethical standards outlined in the Declaration of Helsinki and institutional guidelines for research involving human subjects.

##### Informed Consent

All participants provided informed consent electronically prior to participation. The consent form outlined the purpose of the study, the voluntary nature of participation, the procedures for data handling and anonymity, and the right of participants to withdraw at any time without penalty. Consent was obtained via the Qualtrics platform in the form of ticking a checkbox and was recorded following Ethics Board–approved procedures.

##### Privacy and Confidentiality

Participant data were collected anonymously, with no identifying information retained. IP addresses were not stored, and all responses were deidentified at the point of collection. Data were stored securely on encrypted institutional servers, accessible only to authorized research team members. All analyses were conducted on anonymized datasets, and all reported findings were based on aggregated results.

##### Compensation Details

Participants received monetary compensation for their time, consistent with fair market rates for online panel participation. Compensation was provided by Qualtrics in line with their participant remuneration policies, and participants were informed of compensation prior to consenting to take part in the study.

### Results

#### Face and Content Validity

The face validity of all items was high (mean 3.6). Based on the qualitative feedback, we reworded 2 items to further improve face validity. To assess content validity, we calculated interrater agreement using the Item Content Validity Index, following the threshold of 0.80 recommended by Wynd et al [41]. Items with an Item Content Validity Index value below this threshold were removed (n=6), and 3 additional items were reworded based on expert feedback.

## Study 2

### Methods

#### Participants

A sample of UK residents (n=1037) was recruited through the Qualtrics [42] online research panel, using quota-based convenience sampling. Eligibility criteria included age (18-65 years), location (United Kingdom), and fluency in English. Quotas were applied to align the sample with UK population distributions regarding age, gender, ethnicity, and region, based on 2020 census data and state statistics [43]. This approach ensured demographic diversity reflective of national benchmarks, although the sample was not probabilistically representative in a strict statistical sense. Recruitment was stratified by UK regions (England, Scotland, Wales, and Northern Ireland). Based on quality and eligibility criteria (total survey completion duration less than one-third of the median survey duration, incorrect responses to 1 attention filter question, nonfluency in English, location other than the United Kingdom, and age younger than 18 years or older than 65 years), we removed 17 participants. The final sample included 1020 participants.

#### Measures

The QWI includes 45 items and asks individuals to evaluate their experiences over the past 2 weeks. Participants are presented with the cue “During the past two weeks...” and then with the item (eg, “I have been experiencing stress in my work life”). We opted for a 2-week recall period as it helps minimize recall bias while still capturing meaningful patterns in recent experiences [44,45]. Furthermore, previous research has suggested that a 2-week recall period offers a practical balance between reducing recall bias and capturing a representative summary of well-being, rather than a momentary state [46,47]. This timeframe has been adopted in several validated and widely used mental health questionnaires, including the Warwick-Edinburgh Mental Well-being Scale [17], the World Health Organization-5 Well-being Index [48], the Patient Health Questionnaire-9 [49], and the Generalized Anxiety Disorder-7 [50]. Next, participants rate each item on how frequently each applies to them from “Never” (0 points) to “Always” (6 points). We chose a 7-point Likert scale as it provides better reliability and differentiation than shorter scales [51], reduces acquiescence biases and measurement errors while maintaining cognitive ease [52], and is commonly used in validated well-being scales (World Health Organization-5 [53,54]), allowing for comparability.

Furthermore, frequency-based measures have been increasingly favored in well-being research for their ecological validity and greater clarity in capturing behavioral patterns, compared to agreement-based formats [55,56]. The choice of a 7-point scale balances granularity with usability, providing sufficient sensitivity to detect meaningful differences while maintaining simplicity for workplace administration [51]. The presence of a clear midpoint further supports respondent clarity and reduces cognitive load, particularly in diverse occupational settings [57].

#### Procedure

The survey was built online on Qualtrics. Participants reviewed the research information and gave their consent to take part in the study. Next, participants completed demographic questions and then the QWI items. The consent form and demographic questions were presented in a fixed order, while the items from the QWI and the attention filter were shown in a random order. The median completion duration of the survey was 6 minutes.

#### Statistical Analysis

For exploratory factor analysis (EFA), we used the psych package for R [58]. We used oblimin rotation as we expected the factors to be correlated. We verified the sampling adequacy with the Kaiser-Meyer-Olkin measure and Bartlett test of sphericity. We used different factor extraction criteria such as the scree plot, Kaiser criterion of eigenvalues >1, VSS (very simple structure) criterion [59], and parallel analysis [60]. We removed items with loadings <0.50, uniqueness >0.50, and commonality <0.50.

### Results

#### Sample Characteristics

The final sample consisted of 1020 UK residents (525 female, 51.5%; 1 person did not disclose their gender), ranging in age from 18 to 65 years (mean 43.06, SD 12.98 years). The sample was spread around the United Kingdom (England: 886/1020, 86.8%; Scotland: 77/1020, 7.5%; Wales: 38/1020, 3.8%; Northern Ireland: 19/1020, 1.9%) and was highly educated (tertiary education: 740/1020, 72.5%; secondary education: 276/1020, 27.1%; primary education: 4/1020, 0.4%). In terms of ethnicity, 84.4% (861/1020) identified as White; 5.6% (57/1020) as Asian or Asian British; 7.1% (72/1020) as Black, African, Caribbean, or Black British; 2.5% (25/1020) as mixed; and 0.5% (5/1020) as other ethnic groups. The majority of the sample was in full-time employment (760/1020, 74.5%) and in nonmanagerial positions (627/1020, 61.5%).

#### EFA Results

The Kaiser-Meyer-Olkin value was 0.98, and the Bartlett test of sphericity (*χ*^2^_990_=37063.54; *P*<.001) indicated that correlations between the 45 items were adequately large for the EFA. The scree plot indicated a 4-factor solution, the Kaiser and VSS criteria recommended a 5-factor model, and the parallel analysis suggested a 6-factor solution. We opted for a 5-factor solution as recommended by 2 criteria (Kaiser and VSS criteria) and ran the EFA. When visually inspecting the loadings of the items, we observed that all items loaded on 4 factors. Therefore, we reran the EFA with 4 factors, and the model explained 0.60 of the variance.

We excluded 10 items considering their low loadings, high uniqueness, and low commonality levels. Subsequently, the model underwent refitting with the remaining 35 items. This final iteration, comprising 4 factors, accounted for 64% of the variance, with each factor demonstrating an eigenvalue of 1.

Next, we defined appropriate labels for the 4 factors. The items that loaded on the first factor suggested aspects, such as positive affect, social connectedness, life satisfaction, and mindfulness, related to personal life well-being, and thus, the factor was labeled “thrive and connect in personal life.” The second factor was related to positive affect, social connectedness, and life satisfaction of workplace well-being and was labeled “thrive and connect at work.” The third and fourth factors corresponded to our hypothesized constructs of mind and body and were labeled “mental health” and “physical health,” respectively. Table 1 presents the 4 factors, their items, and their loadings.

**Table 1:** Quan Well-being Index factors, their items, and their loadings.

Quan Well-being Index factors and items			Loading
**Thrive and connect in personal life**			
	1. I have been in a positive mood in my personal life.	0.61	
	2. I have been able to focus on the present moment and accept my feelings and thoughts in my personal life.	0.54	
	3. I have been satisfied with my relationships in my personal life.	0.79	
	4. Support from others has been available to me in my personal life.	0.69	
	5. I have been able to support others in my personal life.	0.61	
	6. I have been feeling appreciated in my personal life.	0.79	
	7. I have been experiencing a sense of belonging in my personal life.	0.86	
	8. I have been feeling satisfied with my personal life.	0.84	
	9. I have been feeling fully engaged in my personal life activities.	0.60	
	10. I have been feeling that I am able to continuously learn, change, and thrive in my personal life.	0.57	
	11. I have been feeling a sense of purpose in my life.	0.58	
	12. I have been feeling that my purpose aligns with my personal actions.	0.50	
	13. I have been feeling that what I do in my personal life is meaningful.	0.74	
	14. I have been feeling optimistic about the future of my personal life.	0.77	
**Thrive and connect at work**			
	15. I have been in a positive mood in my work life.	0.63	
	16. I have been satisfied with my relationships in my work life.	0.65	
	17. Support from others has been available to me in my work life.	0.69	
	18. I have been able to support others in my work life.	0.55	
	19. I have been feeling appreciated in my work life.	0.84	
	20. I have been experiencing a sense of belonging in my work life.	0.84	
	21. I have been feeling satisfied with my work life.	0.86	
	22. I have been feeling fully engaged in my job-related activities.	0.71	
	23. I have been feeling that I am able to continuously learn, change, and thrive in my job.	0.75	
	24. I have been feeling that my purpose aligns with my work activities.	0.66	
	25. I have been feeling that what I do in my organization is meaningful.	0.78	
	26. I have been feeling optimistic about the future of my organization.	0.70	
**Physical health**			
	27. I have been eating and drinking healthily.	0.76	
	28. I have been satisfied with the amount of physical activity I have had.	0.70	
	29. I have been able to manage my cravings without depending on them.	0.73	
	30. I have been feeling good about my body.	0.63	
**Mental health**			
	31. I have been experiencing stress in my personal life.	0.62	
	32. I have been experiencing stress in my work life.	0.69	
	33. I have been feeling nervous, anxious, or on edge in my personal life.	0.67	
	34. I have been feeling nervous, anxious, or on edge in my work life.	0.70	
	35. I have been feeling burned out.	0.55	

## Study 3

### Methods

#### Participants

An independent sample of participants (n=966) was recruited to conduct the confirmatory factor analysis (CFA) via the Qualtrics platform, with the same inclusion criteria as in study 2. To assess test-retest reliability, a subset of participants (n=275) completed the QWI 2 weeks after the CFA stage.

#### Measures and Procedure

##### CFA and Convergent and Divergent Validity

A section of the questionnaire included a demographics survey that was identical to the one used in study 2. Participants completed the 35-item QWI developed in study 2. For convergent validity, the Flourishing-at-Work Scale–Short Form [61] and the Warwick-Edinburgh Mental Well-being Scale [17] were used, and for divergent validity, the Big Five Personality Inventory short version [62] was used. All questionnaires and items were presented in a randomized order. The median completion duration of the survey was 13 minutes.

##### Test-Retest Reliability

For the test-retest analysis, participants completed the 35-item QWI a second time (items presented in a randomized order) approximately 2 weeks after the CFA and convergent and divergent validity stages. The median completion duration of the survey was 3 minutes.

##### Statistical Analysis

The lavaan package for R [63] was used for the CFA. To evaluate the factor structure of the QWI, we tested 4 different factor models. In model 1, we tested a hierarchical model with a general factor (general well-being) that influenced the 4 group factors (thrive and connect in personal life, thrive and connect at work, mental health, and physical health), which in turn affected the items that belong in each of them. In model 2, we explored a transformed variant of the hierarchical model (Schmid-Leiman) in which both the general factor and the 4 group factors directly influenced the items. In model 3, we assessed a simple CFA model, where only the 4 group factors influenced the items, and there was no general factor. In model 4, we tested a similar CFA model as model 3, but allowed intercorrelations between the group factors. All 35 items were loaded on the same factors as suggested by the EFA, and only the factor relation structure differed.

#### Results

##### Sample Characteristics

The final sample consisted of 966 participants, with a mean reported age of 44.4 (SD 12.52) years. Of the 966 participants, 486 (50.3%) were male and 480 (49.7%) were female. Regarding ethnicity, 87.6% (846/966) of participants identified as White; 6.9% (67/966) as Black, African, Caribbean, or Black British; 3.3% (32/966) as Asian or Asian British; 1.9% (18/966) as mixed; and 0.3% (3/966) as other ethnic groups. Regarding region, 88.9% (859/966) of participants were based in England, 6.2% (60/966) in Scotland, 3.6% (35/966) in Wales, and 1.2% (12/966) in Northern Ireland. The majority of the sample was highly educated (tertiary education: 508/966, 52.6%; secondary education: 455/966, 47.1%; primary education: 3/966, 0.3%). The majority of participants were in permanent employment (full-time or part-time work: 786/966, 81.4%), while 14.9% (144/966) were self-employed and 3.7% (36/966) were employed in temporary or contractual work.

In the test-retest reliability subset (n=275), the mean age was 52.12 (SD 9.56) years. Of the 275 participants in this subset, 105 (38.2%) were male and 170 (61.8%) were female. Regarding ethnicity, 93.1% (256/275) of participants identified as White; 3.3% (9/275) as Asian or Asian British; 1.5% (4/275) as Black, African, Caribbean, or Black British; and 2.2% (6/275) as mixed.

##### CFA: Model Testing and Evaluation

The fit indices, model comparison fit indices, and model comparison statistics for all 4 models are presented in Table 2. Model 1 demonstrated the best fit to the data (*χ*^2^_521_=3467.00; Bentler comparative fit index=0.906; Tucker-Lewis index=0.892; root mean square error of approximation=0.077; standardized root mean square residual=0.048) and was superior to all other models in terms of both Akaike information criterion (AIC) and Bayesian information criterion (BIC) values (ΔAIC=0; ΔBIC=0). Models 2, 3, and 4 displayed poorer fit, with substantially higher AIC and BIC values (ΔAIC ≥590.08; ΔBIC ≥531.17). Interestingly, models 3 and 4 yielded identical fit indices (*χ*^2^_554_=4657.33; Bentler comparative fit index=0.869; Tucker-Lewis index=0.859; root mean square error of approximation=0.088; standardized root mean square residual=0.064), suggesting that the addition of interfactor correlations in model 4 did not improve model fit. This finding was likely due to weak or negligible correlations among the group factors.

**Table 2:** Goodness-of-fit statistics comparison results for the 4 structural equation models.

CFA^a^ models	Chi-square (*df*)	CFI^b^	TLI^c^	RMSEA^d^	SRMR^e^	ΔAIC^f^	ΔBIC^g^
Model 1 (hierarchical)	3467.00 (521)	0.906	0.892	0.077	0.048	0.00	0.00
Model 2 (Schmid-Leiman)	4658.26 (556)	0.869	0.859	0.087	0.064	590.08	531.17
Model 3 (simple factor)	4657.33 (554)	0.869	0.859	0.088	0.064	1124.32	1494.68
Model 4 (factor intercorrelation)	4657.33 (554)	0.869	0.859	0.088	0.064	593.15	963.51

^a^CFA: confirmatory factor analysis.

^b^CFI: Bentler comparative fit index.

^c^TLI: Tucker-Lewis index.

^d^RMSEA: root mean square error of approximation.

^e^SRMR: standardized root mean square residual.

^f^ΔAIC: relative difference in the Akaike information criterion between the model and the best model.

^g^ΔBIC: relative difference in the Bayesian information criterion between the model and the best model.

We assessed the internal reliability of the group factors with Cronbach α, McDonald ω total [64], and Guttman λ6 [65]. As presented in Table 3, estimates of internal reliability were good or very good for all the factors.

**Table 3:** Summary statistics of the Quan Well-being Index factors, and indicators of internal reliability (n=966) and test-retest reliability (n=275).

Reliability indicator	Thrive and connect in personal life	Thrive and connect at work	Mental health	Physical health	General well-being
Mean (SD)	51.83 (19.21)	40.57 (18.56)	13.42 (5.83)	18.10 (6.82)	123.92 (41.51)
Maximum	84	72	24	30	219
Minimum	0	0	0	0	9
Cronbach α	.96	.97	.86	.85	.97
McDonald ω total	0.96	0.97	0.86	0.85	0.97
Guttman λ6	0.96	0.97	0.86	0.82	0.98
Test-retest correlation	0.81	0.74	0.74	0.80	0.81

##### Convergent and Divergent Validity

Table 4 presents the correlations of the 5 QWI factors with all the questionnaires that were implemented for convergent and divergent validity. The convergent validity of the QWI can be seen in the strong correlations of all 5 factors with the Warwick-Edinburgh Mental Well-being Scale (*r*=0.45 to 0.85), with the highest value obtained for the general well-being factor (*r*=0.85). Further contributing to the convergent validity of the QWI are the very strong correlations between the “thrive and connect at work” factor of the QWI and all the Flourishing-at-Work Scale factors (*r*=0.80 to 0.84). Divergent validity was established by the weak and negative correlations between all QWI factors and all Big Five Personality Inventory factors.

**Table 4:** Pearson correlation coefficients between Quan Well-being Index factors and existing measures of well-being in personal life and at the workplace, and personality traits.

Existing measures			Thrive and connect in personal life		Thrive and connect at work		Mental health		Physical health		General well-being
Warwick-Edinburgh Mental Well-being Scale			0.82		0.67		0.45		0.68		0.85
**Flourishing-at-Work Scale**											
	Emotional well-being	0.57		0.80		0.27		0.48		0.73	
	Psychological well-being	0.54		0.82		0.16		0.46		0.71	
	Social well-being	0.54		0.84		0.21		0.47		0.72	
**Big Five Personality Inventory**											
	Extraversion	0.10		0.11		0.04		0.02		0.10	
	Agreeableness	0.30		0.33		0.17		0.26		0.35	
	Conscientiousness	0.27		0.26		0.19		0.27		0.31	
	Neuroticism	–0.46		–0.38		–0.48		–0.44		–0.52	
	Openness to experience	0.09		0.04		–0.02		0.06		0.06	

##### Test-Retest Reliability

Table 3 presents the test-retest correlations for the 5 factors, which were determined to be strong to very strong (ranging from 0.74 to 0.81) and statistically significant (*P*<.001).

**Figure 1:**
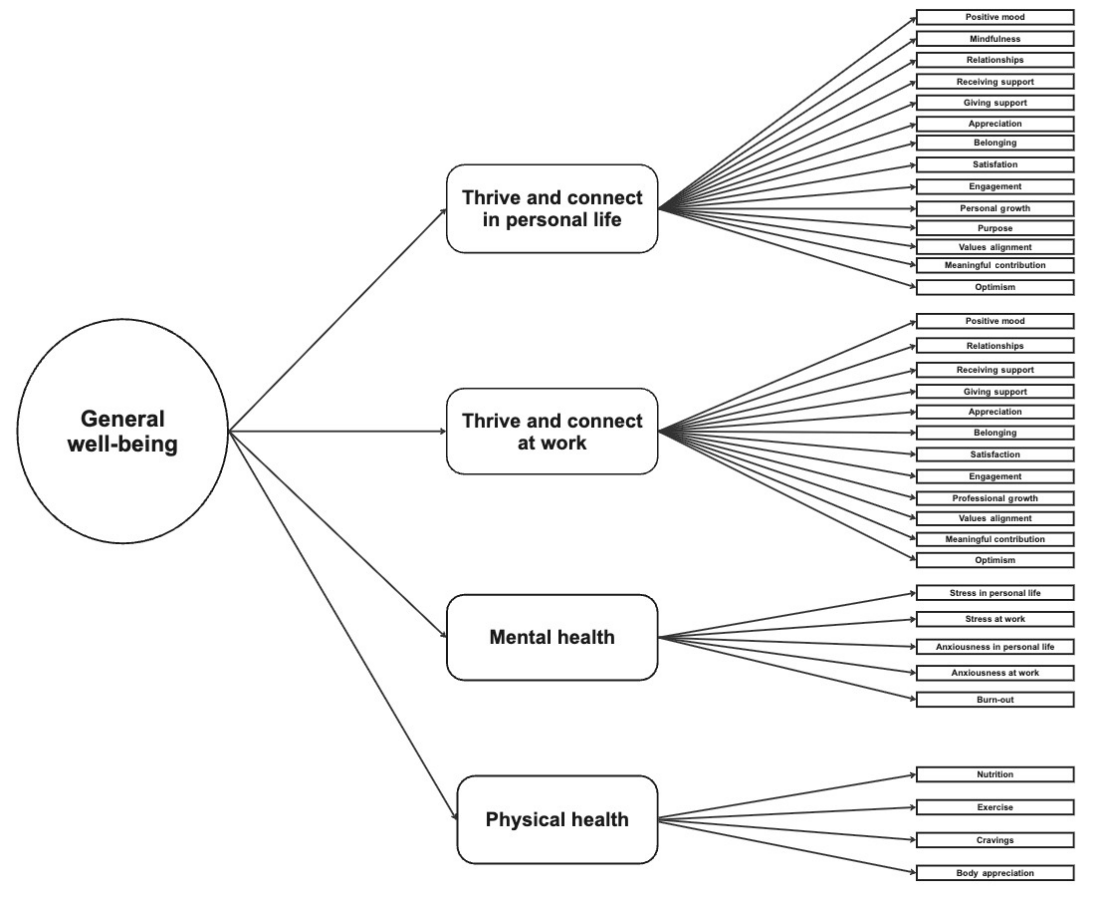
The factor structure of the Quan Well-being Index as demonstrated by model 1 (hierarchical model) in the confirmatory factor analysis.

Overall, the results from study 3 indicated that model 1 (Figure 1) was the best-fitting model, supporting the hypothesized hierarchical structure where the “general well-being” factor influenced the 4 group factors, which in turn impacted their associated individual items. The Bentler comparative fit index, the observed reliability coefficients, and the correlations with the additional questionnaires that were used indicate that the 35-item QWI has the required level of reliability and validity.

## Discussion

### Overview

We report on the development of the QWI, a novel self-report index that measures well-being in personal life and at the workplace. The factors identified in this index encompass, corroborate, and quantify aspects of well-being that have previously only been examined in isolation, integrating them into a single measurement instrument for the first time [39,55]. The development of QWI factors has also generated new insights into well-being in personal life and at the workplace, which are presented below.

### Summary of Key Findings

The development and validation of the QWI as a novel and holistic measure of well-being in personal life and at the workplace have been demonstrated through a series of studies, showing good internal validity, convergent and divergent validity, and test-retest reliability. This comprehensive tool encompasses 5 key factors that collectively provide a holistic well-being assessment.

The first factor “thrive and connect in personal life” covers various aspects of personal life, including emotional state, mindfulness, relationships, social support, engagement, and sense of purpose. These results indicate that well-being in personal life is a multifaceted concept, encompassing various interconnected elements that contribute to an individual’s overall life satisfaction and psychological health [39,66]. The second factor “thrive and connect at work” covers various aspects of work life, such as emotions, relationships, personal growth, and feeling valued, aligning with prior research emphasizing the importance of workplace well-being [26,31]. The third factor “mental health” emphasizes the need to consider mental health issues in both personal and professional contexts, recognizing that individuals may experience different levels of feelings (eg, stress) in personal life and at the workplace [66]. The fourth factor “physical health” is an essential aspect of holistic well-being that covers characteristics related to physical activity and nutrition, consistent with research highlighting the interdependence of physical and psychological health [40,67].

### Theoretical and Practical Implications

The presence of a general well-being factor above the 4 group factors (*thrive and connect in personal life*, *thrive and connect at work*, *physical health*, and *mental health*) suggests that well-being is best conceptualized as a higher-order, integrative construct that unites experiences across both personal and professional life domains. This supports the growing body of research emphasizing the systemic and interrelated nature of well-being across life domains [68,69].

Theoretically, this structure resonates with recent multidimensional and ecological models of well-being [70,71], which move beyond binary hedonic or eudaimonic frameworks and instead frame well-being as a dynamic model influenced by physical, psychological, social, and contextual factors. The QWI’s higher-order structure reflects this complexity, recognizing that individual well-being outcomes do not occur in isolation but are shaped by conditions both at the workplace and in personal life.

Furthermore, the finding that *thrive and connect in personal life* and *thrive and connect at work* load onto a common higher-order factor challenges the often rigid boundaries between occupational and personal well-being that dominate traditional models. This aligns with the “whole person” perspective emerging in organizational psychology [72,73], which posits that work and nonwork experiences are mutually influential. For instance, positive interpersonal dynamics at work can buffer stress in personal life, while strong personal relationships may enhance workplace engagement and mood [67].

QWI’s hierarchical structure is broadly consistent with prominent multidimensional models of well-being, including the PERMA model [39], which conceptualizes well-being as a composite of positive emotion, engagement, relationships, meaning, and accomplishment. However, the QWI makes a distinctive contribution by operationalizing both domain-specific well-being (*thrive and connect at work* vs *thrive and connect in personal life*) and foundational health factors (*physical health* and *mental health*) within a single integrated measurement framework. This dual focus on “where” well-being is experienced and “how” it is sustained may extend existing models by offering a more pragmatic approach for organizational interventions.

The presence of the “thrive and connect” concept as a factor both in personal life and at the workplace indicates that there is a need for distinction between aspects that take place in different settings. As such, individuals are provided with a tool to track and take action on their well-being in separate contexts, which might require different approaches. For example, issues related to work could be brought up in one-to-one meetings between employees and their managers to facilitate bidirectional communication about aspects that might not be apparent in one’s work but can be adjusted to promote healthier organizations [74]. Moreover, the ability to differentiate between personal and professional well-being factors could lead to more targeted interventions and support systems both at the individual level and within organizational structures, ultimately contributing to a more holistic approach to well-being management.

From a practical standpoint, the presence of a general well-being factor allows for both macro- and micro-level analysis. At the macro level, the general factor serves as a useful indicator of an individual’s or team’s holistic well-being, enabling organizations to track broad shifts over time or following interventions. At the micro level, the 4 factors facilitate precise diagnostics, allowing leaders or practitioners to identify specific areas (eg, workplace social connectedness vs mental health) that require tailored support.

Scoring is structured to align with the hierarchical factor model validated in study 3. Each item contributes to 1 of 4 factors: *thrive and connect in personal life*, *thrive and connect at work*, *physical health*, and *mental health*. Factor scores are calculated by averaging responses to the items that belong to each factor, and the overall QWI score is the average of the 4 factors. For interpretative purposes in applied settings, we offer the following score ranges: scores of 0.0-1.9 suggest low well-being and may indicate risk, scores of 2.0-3.9 reflect moderate well-being, and scores of 4.0-6.0 indicate high well-being or “thriving.” These guidelines are intended to facilitate meaningful interpretation and intervention planning by researchers, practitioners, and organizational stakeholders.

### Comparison With Existing Measures of Well-Being

A key contribution of the QWI lies in its integrated assessment of both the personal and professional domains of well-being within a single, psychometrically validated instrument. While several established measures of well-being exist, such as the Warwick-Edinburgh Mental Well-being Scale [17], World Health Organization-5 Well-being Index [54], and PERMA Profiler [75], these tend to focus on general mental well-being and positive functioning, often without capturing the complex interplay between work and nonwork settings.

The QWI addresses this gap by explicitly incorporating 4 interrelated dimensions: *thrive and connect in personal life*, *thrive and connect at work*, *physical health*, and *mental health*. This structure allows for the simultaneous assessment of cross-domain experiences and provides a more ecologically valid representation of how individuals experience well-being across the life course. In contrast, measures like the Job Satisfaction Survey [76] or the Utrecht Work Engagement Scale [77] focus narrowly on occupational well-being, whereas personal well-being tools, such as the Ryff Psychological Well-being Scales [78], do not account for work-related dynamics.

From a practical standpoint, the QWI is designed for ease of use in organizational settings. It comprises 35 items, takes approximately 5 to 7 minutes to complete, and uses a uniform 7-point Likert response scale ranging from “Never” (0 points) to “Always” (6 points). This balance between comprehensiveness and brevity makes it well suited for routine monitoring in time-constrained environments, a feature less common in longer instruments like Ryff scales (84 items) or multidimensional surveys requiring separate administration for work and nonwork domains.

Moreover, by capturing personal and professional well-being within 1 coherent framework, the QWI reduces the burden on respondents and provides practitioners with an integrated profile that enhances intervention planning. This is particularly advantageous in applied settings where holistic well-being strategies (eg, action planning, leadership feedback, and human resource initiatives) benefit from a unified data source. However, we acknowledge that combining domains may reduce granularity in highly specialized research contexts, which remains a consideration when choosing between integrated and domain-specific tools.

Taken together, the QWI’s content coverage, time-efficient administration, and psychometric strength position it as a novel and practical addition to the well-being assessment landscape, particularly for contexts requiring cross-domain insights and targeted, team-level interventions.

### Limitations and Future Directions

Certain limitations and future directions that stemmed from the outcomes of this research need to be addressed. The United Kingdom–based sample we recruited may limit the generalizability of the findings to other cultures, for example, non-Western cultures [79]. Validation in other countries might require the inclusion or exclusion of certain items to support different conceptualizations of well-being [80]. For example, in countries that have indigenous populations, such as New Zealand and Australia, items that represent family, land, and rituals might be necessary to encompass their well-being experiences [81]. We are planning to translate and validate the QWI for other continents as well as countries across Europe to explore how well it maps in other languages and what further improvements need to be made. In doing so, particular care will be taken to ensure conceptual, linguistic, and metric equivalence across cultural contexts, which may include exploratory and confirmatory factor analyses in each new language group.

Additionally, although the QWI demonstrated good convergent validity with established well-being measures, all data were derived from self-report instruments. This mono-method approach may introduce shared method variance and raise concerns about response biases, including social desirability and acquiescence effects [82]. To strengthen construct validity and provide a more holistic view of well-being, future studies should incorporate a multimethod approach. This may include behavioral data (eg, absenteeism rates or job performance metrics) and objective physiological indicators, such as cortisol levels, heart rate variability, and sleep quality measures, which can serve as indirect proxies for stress and overall health.

Lastly, while the 7-point Likert response format (ranging from “Never” to “Always”) was chosen based on evidence suggesting that it improves reliability and reduces response bias, it may not be optimal in all cultural or linguistic contexts. Adaptations to the scale’s format may be necessary when validating the QWI globally, particularly in regions where numeric or verbal anchors are interpreted differently due to linguistic or cultural norms.

By addressing these limitations, future research will be able to further assess the utility, adaptability, and impact of the QWI as a cross-culturally valid and practically useful tool for measuring employee well-being across personal and professional life domains.

### Conclusion

In summary, the QWI is a novel, reliable, and validated instrument that systematically measures multiple and distinct aspects of well-being in personal life and at the workplace. It is anticipated that the QWI will be valuable to digitally assess well-being, enabling individuals, teams, and organizations to gain insights and monitor their progress over time, implement appropriate interventions, and ultimately enhance well-being areas that require improvement.

## Abbreviations

AIC: Akaike information criterion
BIC: Bayesian information criterion
CFA: confirmatory factor analysis
EFA: exploratory factor analysis
JD-R: Job Demands-Resources
PERMA: positive emotion, engagement, relationships, meaning, and accomplishment
QWI: Quan Well-being Index
SDT: Self-Determination Theory
VSS: very simple structure

## Data Availability

The Quan Well-being Index (QWI) is freely available for academic research purposes. Researchers interested in using the QWI in scholarly studies are encouraged to contact the corresponding author to request access to the full instrument and accompanying scoring guidelines. For commercial or applied use in organizational settings, the QWI is proprietary and forms part of the well-being assessment platform developed by Quan BV. Commercial entities or practitioners seeking to implement the QWI in workplace interventions should contact the authors or Quan BV to discuss licensing or access terms.

## References

[1] Hassard J, Thomson L, Blake H, Day A, Cooper CL (2023). Understanding and Exploring the Cost of Poor Mental Health at Work for Organizations and Society. The Routledge Companion to Mental Health at Work.

[2] Pinheiro M, Ivandic I, Razzouk D, Razzouk D (2017). The Economic Impact of Mental Disorders and Mental Health Problems in the Workplace. Mental Health Economics.

[3] Lu L, Day A, Cooper CL (2023). Balancing Performance and Well-Being: Motivational and Resource Regulation in the Context of Excessive Availability for Work. The Routledge Companion to Mental Health at Work.

[4] Harshita L, Senthil A (2021). Impact of employee well-being on organizational performance in workplace. Wesleyan J Res.

[5] Cooper CL, Cartwright S (1994). Healthy mind; healthy organization: a proactive approach to occupational stress. Human Relations.

[6] Goetzel R, Roemer E, Holingue C, Fallin M, McCleary K, Eaton W, Agnew J, Azocar F, Ballard D, Bartlett J, Braga M, Conway H, Crighton KA, Frank R, Jinnett K, Keller-Greene D, Rauch SM, Safeer R, Saporito D, Schill A, Shern D, Strecher V, Wald P, Wang P, Mattingly CR (2018). Mental health in the workplace: a call to action proceedings from the mental health in the workplace-public health summit. J Occup Environ Med.

[7] (2022). Guidelines on mental health at work. World Health Organization.

[8] Stevenson D, Farmer P (2017). Thriving at Work: a review of mental health and employers. GOV.UK.

[9] Pohl M (2017). 325,000 mobile health apps available in 2017 – Android now the leading mHealth platform. Research2Guidance.

[10] Fleming WJ (2024). Employee well‐being outcomes from individual‐level mental health interventions: Cross‐sectional evidence from the United Kingdom. Industrial Relations Journal.

[11] (2019). WHO guideline: recommendations on digital interventions for health system strengthening. World Health Organization.

[12] (2017). Report of the Working Group on mHealth assessment guidelines. European Commission.

[13] Magrabi F, Habli I, Sujan M, Wong D, Thimbleby H, Baker M, Coiera E (2019). Why is it so difficult to govern mobile apps in healthcare?. BMJ Health Care Inform.

[14] Lau N, O'Daffer A, Colt S, Yi-Frazier JP, Palermo TM, McCauley E, Rosenberg AR (2020). Android and iPhone mobile apps for psychosocial wellness and stress management: systematic search in app stores and literature review. JMIR Mhealth Uhealth.

[15] Azzone V, McCann B, Merrick EL, Hiatt D, Hodgkin D, Horgan C (2009). Workplace stress, organizational factors and EAP utilization. J Workplace Behav Health.

[16] Cooke P, Melchert T, Connor K (2016). Measuring well-being: A review of instruments. The Counseling Psychologist.

[17] Tennant R, Hiller L, Fishwick R, Platt S, Joseph S, Weich S, Parkinson J, Secker J, Stewart-Brown S (2007). The Warwick-Edinburgh Mental Well-being Scale (WEMWBS): development and UK validation. Health Qual Life Outcomes.

[18] Frisch MB, Cornell J, Villanueva M, Retzlaff PJ (1992). Clinical validation of the Quality of Life Inventory. A measure of life satisfaction for use in treatment planning and outcome assessment. Psychological Assessment.

[19] Balogh P, Krasz KG, Kun (2016). Development of the work-related well-being questionnaire based on Seligman’s PERMA model. Period Polytech Soc Man Sci.

[20] Orsila R, Luukkaala T, Manka M, Nygard C (2011). A new approach to measuring work-related well-being. Int J Occup Saf Ergon.

[21] Parker G, Hyett M (2011). Measurement of well-being in the workplace: the development of the work well-being questionnaire. J Nerv Ment Dis.

[22] Leiter MP, Maslach C, Tetrick LE, Fisher GG, Ford MT, Quick JC (2024). Job burnout. Handbook of Occupational Health Psychology.

[23] Stanton JM, Balzer WK, Smith PC, Parra LF, Ironson G (2001). A general measure of work stress: the stress in general scale. Educational and Psychological Measurement.

[24] van Saane N, Sluiter J, Verbeek J, Frings-Dresen M (2003). Reliability and validity of instruments measuring job satisfaction--a systematic review. Occup Med (Lond).

[25] Taris TW, Schaufeli WB, Cooper C (2018). Individual Well-Being and Performance at Work: A conceptual and theoretical overview. Current Issues in Work and Organizational Psychology.

[26] Bakker AB, Demerouti E (2007). The Job Demands-Resources model: State of the art. Journal of Managerial Psychology.

[27] Bakker AB, Demerouti E, Sanz-Vergel A (2023). Job demands–resources theory: ten years later. Annu Rev Organ Psychol Organ Behav.

[28] Hartmann S, Weiss M, Newman A, Hoegl M (2019). Resilience in the workplace: a multilevel review and synthesis. Applied Psychology.

[29] Tetrick LE, Winslow CJ (2015). Workplace stress management interventions and health promotion. Annu Rev Organ Psychol Organ Behav.

[30] Xanthopoulou D, Bakker AB, Demerouti E, Schaufeli WB (2007). The role of personal resources in the job demands-resources model. International Journal of Stress Management.

[31] Ryan RM, Deci EL (2000). Self-determination theory and the facilitation of intrinsic motivation, social development, and well-being. Am Psychol.

[32] Gagné M, Deci EL (2005). Self‐determination theory and work motivation. J Organ Behavior.

[33] Deci EL, Connell JP, Ryan RM (1989). Self-determination in a work organization. Journal of Applied Psychology.

[34] Van den Broeck A, Ferris DL, Chang C, Rosen CC (2016). A review of self-determination theory’s basic psychological needs at work. Journal of Management.

[35] Baard PP, Deci EL, Ryan RM (2006). Intrinsic need satisfaction: a motivational basis of performance and well-being in two work settings. J Applied Social Pyschol.

[36] Kauer SD, Reid SC, Crooke AHD, Khor A, Hearps SJC, Jorm AF, Sanci L, Patton G (2012). Self-monitoring using mobile phones in the early stages of adolescent depression: randomized controlled trial. J Med Internet Res.

[37] Wichers M, Simons C, Kramer I, Hartmann J, Lothmann C, Myin-Germeys I, van Bemmel AL, Peeters F, Delespaul PH, van Os J (2011). Momentary assessment technology as a tool to help patients with depression help themselves. Acta Psychiatr Scand.

[38] Truong H, McLachlan CS (2022). Analysis of start-up digital mental health platforms for enterprise: opportunities for enhancing communication between managers and employees. Sustainability.

[39] Seligman MEP (2011). Flourish: A Visionary New Understanding of Happiness and Well-being.

[40] Keyes CLM (2007). Promoting and protecting mental health as flourishing: a complementary strategy for improving national mental health. Am Psychol.

[41] Wynd CA, Schmidt B, Schaefer MA (2003). Two quantitative approaches for estimating content validity. West J Nurs Res.

[42] Qualtrics.

[43] Eurostat.

[44] Bradburn N, Rips L, Shevell S (1987). Answering autobiographical questions: the impact of memory and inference on surveys. Science.

[45] Sudman S, Bradburn NM (1973). Effects of time and memory factors on response in surveys. Journal of the American Statistical Association.

[46] Broderick JE, Schwartz JE, Shiffman S, Hufford MR, Stone AA (2009). Recall bias in reporting mental health symptoms: an experience sampling study. J Psychiatr Res.

[47] Shiffman S, Stone AA, Hufford MR (2008). Ecological momentary assessment. Annu Rev Clin Psychol.

[48] Bech P, Gudex C, Johansen KS (1996). The WHO (Ten) Well-Being Index: validation in diabetes. Psychother Psychosom.

[49] Kroenke K, Spitzer RL, Williams JB (2001). The PHQ-9: validity of a brief depression severity measure. J Gen Intern Med.

[50] Spitzer RL, Kroenke K, Williams JBW, Löwe B (2006). A brief measure for assessing generalized anxiety disorder: the GAD-7. Arch Intern Med.

[51] Preston CC, Colman AM (2000). Optimal number of response categories in rating scales: reliability, validity, discriminating power, and respondent preferences. Acta Psychol (Amst).

[52] Finstad K (2010). The usability metric for user experience. Interacting with Computers.

[53] (1998). Wellbeing measures in primary health care/the DepCare Project. World Health Organization.

[54] Topp CW, Østergaard SD, Søndergaard S, Bech P (2015). The WHO-5 Well-Being Index: a systematic review of the literature. Psychother Psychosom.

[55] Diener E, Lucas RE, Oishi S, Snyder CR, Lopez SJ (2018). Subjective Well-Being The Science of Happiness and Life Satisfaction. Handbook of Positive Psychology.

[56] Goodman FR, Disabato DJ, Kashdan TB, Kauffman SB (2017). Measuring well-being: A comparison of subjective well-being and PERMA. The Journal of Positive Psychology.

[57] Weijters B, Baumgartner H, Schillewaert N (2013). Reversed item bias: an integrative model. Psychol Methods.

[58] Package ‘psych’. R Project.

[59] Revelle W, Rocklin T (1979). Very simple structure: an alternative procedure for estimating the optimal number of interpretable factors. Multivariate Behav Res.

[60] Horn JL (2025). A rationale and test for the number of factors in factor analysis. Psychometrika.

[61] Rautenbach C, Rothmann S (2017). Psychometric validation of the Flourishing-at-Work Scale – Short Form (FWS-SF): Results and implications of a South African study. Journal of Psychology in Africa.

[62] Rammstedt B, John OP (2007). Measuring personality in one minute or less: A 10-item short version of the Big Five Inventory in English and German. Journal of Research in Personality.

[63] Rosseel Y (2012). lavaan: An R package for structural equation modeling. J Stat Soft.

[64] Zinbarg RE, Revelle W, Yovel I, Li W (2025). Cronbach’s α, Revelle’s β, and Mcdonald’s ωH: their relations with each other and two alternative conceptualizations of reliability. Psychometrika.

[65] Guttman L (2025). A basis for analyzing test-retest reliability. Psychometrika.

[66] Diener E (2000). Subjective well-being: The science of happiness and a proposal for a national index. American Psychologist.

[67] Sonnentag S (2018). The recovery paradox: Portraying the complex interplay between job stressors, lack of recovery, and poor well-being. Research in Organizational Behavior.

[68] How's Life?. Organisation for Economic Co-operation and Development.

[69] VanderWeele TJ (2017). On the promotion of human flourishing. Proc Natl Acad Sci USA.

[70] Dodge R, Daly A, Huyton J, Sanders L (2012). The challenge of defining wellbeing. Intnl J Wellbeing.

[71] Huppert FA, So TTC (2013). Flourishing across Europe: application of a new conceptual framework for defining well-being. Soc Indic Res.

[72] Grant AM (2007). Relational job design and the motivation to make a prosocial difference. AMR.

[73] Nielsen K, Nielsen MB, Ogbonnaya C, Känsälä M, Saari E, Isaksson K (2017). Workplace resources to improve both employee well-being and performance: A systematic review and meta-analysis. Work & Stress.

[74] Penedo FJ, Dahn JR (2005). Exercise and well-being: a review of mental and physical health benefits associated with physical activity. Curr Opin Psychiatry.

[75] Butler J, Kern ML (2016). The PERMA-Profiler: A brief multidimensional measure of flourishing. Intnl. J. Wellbeing.

[76] Spector PE (1985). Measurement of human service staff satisfaction: Development of the Job Satisfaction Survey. American J of Comm Psychol.

[77] Schaufeli WB, Salanova M, González-Romá V, Bakker AB (2002). The measurement of engagement and burnout: A two sample confirmatory factor analytic approach. J Happiness Stud.

[78] Ryff CD (1989). Happiness is everything, or is it? Explorations on the meaning of psychological well-being. Journal of Personality and Social Psychology.

[79] Scully D, Kremer J, Meade MM, Graham R, Dudgeon K (1998). Physical exercise and psychological well being: a critical review. Br J Sports Med.

[80] Bakker AB, Demerouti E (2017). Job demands-resources theory: Taking stock and looking forward. J Occup Health Psychol.

[81] Henrich J, Heine SJ, Norenzayan A (2010). The weirdest people in the world?. Behav Brain Sci.

[82] Joshanloo M, Van de Vliert E, Jose PE, Kern ML, Wehmeyer ML (2021). Four Fundamental Distinctions in Conceptions of Wellbeing Across Cultures. The Palgrave Handbook of Positive Education.

